# Biomass Bottom Ash as Supplementary Cementitious Material: The Effect of Mechanochemical Pre-Treatment and Mineral Carbonation

**DOI:** 10.3390/ma15238357

**Published:** 2022-11-24

**Authors:** Lorena Skevi, Vahiddin Alperen Baki, Yanjin Feng, Maria Valderrabano, Xinyuan Ke

**Affiliations:** Department of Architecture and Civil Engineering, University of Bath, Bath BA2 7AY, UK

**Keywords:** low-clinker cement, industrial wastes, mechanochemical activation, mineral carbonation

## Abstract

The need to mitigate the CO_2_ emissions deriving from the cement industry becomes imperative as the climate crisis advances. An effective strategy to achieve this is increasing the replacement level of cement clinkers by waste-derived supplementary cementitious materials (SCMs). In this study, the use of mechanochemically activated biomass ash for high-volume (up to 40%) substitution of cement is investigated. The effect of mineral carbonation treatment on the performance of the mechanochemically treated biomass ash as SCM was also examined. The results showed that the mechanochemically treated biomass ash was the most effective SCM, with the respective samples at 40% cement replacement reaching 63% of the strength at 28 days as compared to samples with 100% Portland cement, while only 17% of the strength was achieved in samples with 40% untreated biomass ash. As suggested by the isothermal calorimetry, XRD, FTIR, and TG analysis, the mechanochemical treatment enhanced the reactivity and the filler effect of the biomass ash, leading to improved mechanical performances of these mortars compared to those containing untreated biomass ash. Mineral carbonation reduced the reactivity of the mechanochemically treated biomass ash but still led to better strength performances in comparison to the untreated biomass ash.

## 1. Introduction

The growing expansion of urbanisation over the past century led to extensive use of cement-based materials, raising the cement production to 4.3 Gt globally in 2021 [1]. Currently, 0.59 Gt of CO_2_ is emitted per Gt of cement [1], making the cement industry a major CO_2_ emitter, responsible for about 27% of the total industry-derived [2,3] and 8% of the total anthropogenic CO_2_ emissions [4,5]. Recently, clear goals for the decarbonisation of cement industry have been set, and the net zero emissions scenario set for 2050 has been adopted by policy makers, producers, and international associations in this field [1]. The use of supplementary cementitious materials (SCMs) to produce blended cements (clinker substitution) is one of the main strategies promoted in this direction [1,6]. At the same time, the integration of carbon capture, utilisation, and storage (CCUS) technologies into construction materials plays an important role in achieving the CO_2_ mitigation goals [1,6].

Some commonly used SCMs are industrial byproducts, such as fly ash deriving from the coal combustion in coal-fired power plants [7], ground granulated blast-furnace slag (GGBFS) deriving from steel production [8], and silica fume produced during ferrosilicon alloy production [9]. Depending on their chemical composition and physical properties, as well as the level of substitution, SCMs can act in different ways in the cementitious composite. SCMs contain high reactive aluminosilicate content (i.e., GGBFS) and may have weak/latent hydraulic properties—producing cementitious-like hydration products when in contact with water—and/or pozzolanic properties, reacting with portlandite to produce calcium silicate hydrates [10,11]. SCMs can also act as fillers, allowing more space for hydration products to form and providing at the same time additional nucleation sites for their formation [12]. Thus, when added at certain replacement levels, SCMs can compensate for the dilution effect caused by the lower clinker content [11], resulting in composites with acceptable and even enhanced properties. Silica fume, fly ash, and GGBFS can replace cement up to 10–15%, 20% [7], and 50% [8], respectively, without significant adverse effects. However, the supply of these industrial byproducts is low compared to the cement production rate and is expected to further decline in the years to come as the respective industries shift to more sustainable production routes [13].

The utilization of biomass in power plants, on the other hand, is continuously growing; at present, biomass accounts for 10% of the global energy supply, hence being one of the largest renewable energy sources globally [14,15]. However, environmental concerns over the residues produced during biomass combustion arose from this process. Two types of biomass ashes are produced; biomass fly ash (BFA), which is the fine and lightweight fraction that is transported by flue gases, and biomass bottom ash (BBA), which is the heavier and coarser slag comprised of sand, inorganic components, and unburnt particles [15,16]. To date, biomass ash is mostly disposed of via landfills, imposing risks on both the environment and human health [15,17]. To mitigate this risk, the immobilization of biomass ash in cementitious materials, valorised as SCM, is a promising solution and has been investigated in various studies [18,19,20,21,22,23,24,25,26,27,28,29,30,31,32,33,34,35]. The behaviour of biomass ash as SCMs depends significantly on their physicochemical properties, which are affected by the combustion process (type of feedstock biomass, temperatures used during combustion, method of collection and storage) [17,36,37]. The variation of the biomass ash composition has led to different observations regarding their effects on the mechanical performances and physical properties of cementitious materials across the literature. Several studies reported a strength decline upon the addition of biomass ash, which was pronounced as the substitution level increased [18,19,21,24,25,26,27,29,30,32,34,35]. Different optimum substitution levels, mainly 5% [18,27], 10% [19,20,21,30,31,34], 15% [19,23,25,26,32,35], and 20% [22], have been recommended for imposing a positive effect on the strength of BA cementitious composites. At these levels of substitution, the filling effect of the biomass ash, as well as its potential hydraulic and/or pozzolanic activity, has been found to counteract the dilution effect, resulting in adequate or enhanced strength performances. Pre-treatment of the biomass ash—including water washing [24,34], mechanical and chemical activation [23,38], heating, and removal of organic matter [29]—has been proven effective for improving the reactivity of the ash and thus the mechanical performance of biomass-ash-modified cementitious composites. It should be noted that the majority of the abovementioned studies concerned the utilization of BFA [18,22,23,24,25,26,27,28,31,33,34,35] in cement composites, and only a few focused on BBA [18,20,29,30,39].

Apart from their use as SCMs, alkaline solid wastes in general [40,41,42,43,44] and biomass ash specifically [45] have been investigated as potential materials for carbon sequestration due to their intrinsic alkalinity and high reactivity. Under certain conditions, CO_2_ can be dissolved and then trapped in these materials in the form of stable carbonate minerals or dissolved bicarbonate ions [46]. The efficiency of the sequestration depends heavily on the composition of the available wastes. The presence of phases such as free lime and portlandite in the materials favours the binding of CO_2_ through the carbonation reaction, whereas silicate-rich materials are more difficult to carbonate [43,44]. The lower particle size of the material also results in higher CO_2_ uptake [47]. The minerals’ carbonation is mainly performed in two ways: the direct route, which involves only the step of carbonation, and the indirect, which includes the extra step of Ca extraction prior to the carbonation [44]. The simpler route of direct carbonation often requires high energy input, demanding elevated temperature and/or pressure in the presence of high CO_2_ concentration [48,49,50]. At the moment, the few studies that investigated a low-energy carbonation process are focused on coal fly ash [51,52]. The potential application of low-energy and low-cost mineral carbonation in other alkaline solid wastes, such as biomass ash, could encourage the adoption of such processes. The utilisation of the carbonated products in concrete, partially replacing cement clinkers, could offer further sustainability benefits, leading to net zero cement-based materials.

An emerging method for increasing the chemical reactivity of materials is mechanochemical activation, during which the applied mechanical energy imposes changes on the crystalline structure and the surface properties of the materials, enhancing their reactivity [53,54]. The method has been applied both to improve the reactivity of pozzolanic materials [55,56] and to increase the CO_2_ uptake [53,57] of the mechanochemically activated minerals. An increase in amorphization and the breakage of Si-O and Al-O bonds during the process have been reported, creating additional surfaces and active sites on the surfaces of the solid materials to promote reactivities [58].

In this study, mechanochemical activation is used as a pre-treatment method of a timber BBA, aiming to increase the reactivity and CO_2_ binding capacity of the material. The effects of the mechanochemical treatment on the reactivity of the BBA, with and without follow-on mineral carbonation, are investigated regarding their use as SCMs at different cement substitution levels. The combining benefits of waste utilisation, CO_2_ sequestration, and reduction of cement clinker content are compared and evaluated.

## 2. Materials and Methods

### 2.1. Raw Materials

**Figure 1:**
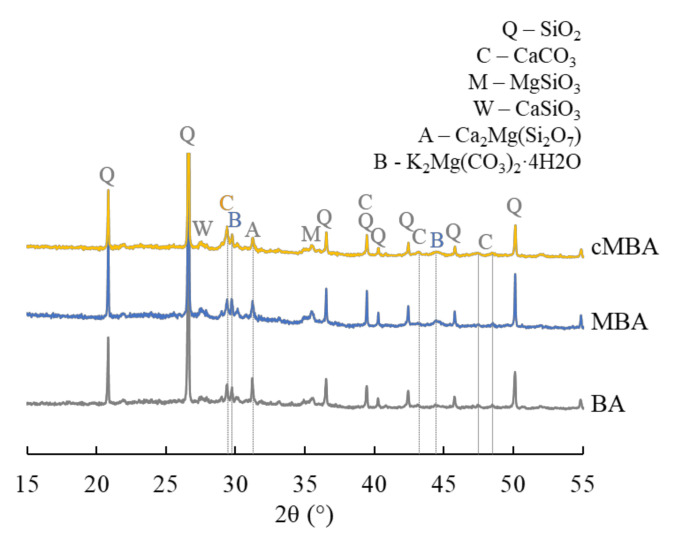
XRD spectra of the biomass ash used in this study as received (BA), after the mechanochemical activation (MBA), and after the mechanochemical and carbonation treatment (cMBA). Q: quartz, C: calcite, M: magnesium silicate, W: wollastonite, A: akermanite, B: baylissite.

**Figure 2:**
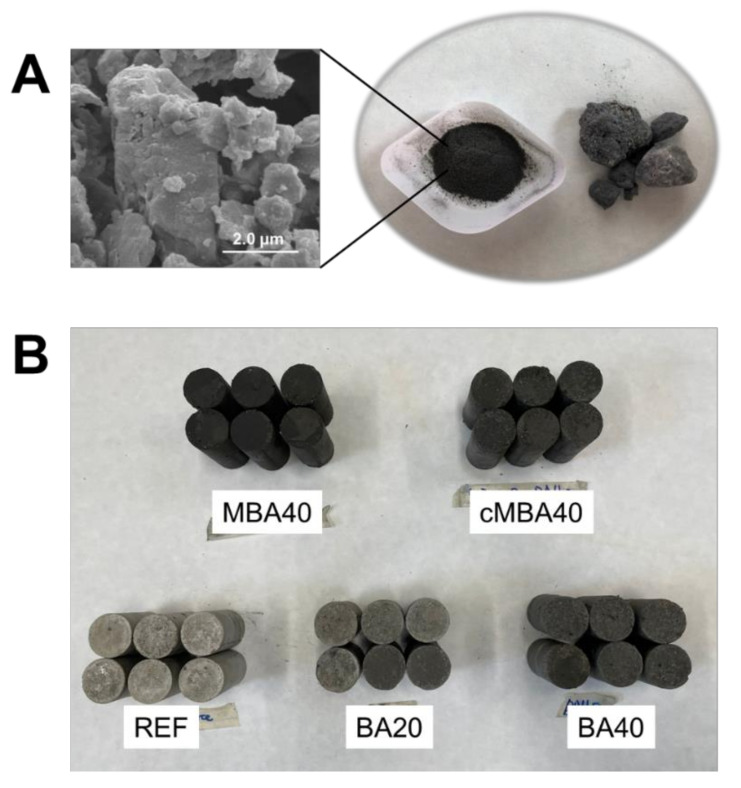
(**A**) SEM image and photo of manually crushed and as-received biomass ash investigated in this study. (**B**) Photo of the mortar cylinders prepared for mechanical testing containing untreated (BA), mechanochemically treated (MBA) and carbonated mechanochemically treated (MBA) biomass at 20% and 40% cement replacement levels, or with no biomass ash addition (REF).

The BBA used in this study is the byproduct of wood timber combustion taking place in an incinerator that operates at 700–1000 °C. After drying at 105 °C, the biomass ash was hand-ground so that it passed the 0.5 mm aperture sieve. The chemical composition of the received biomass ash was comprised of 22% CaO + 4% of other alkalis (MgO + K_2_O + Na_2_O), 45% SiO_2_, 9% Al_2_O_3_, and 8.6% Fe_2_O_3_; LOI was found to be 2.6%, 1.3% of which derived from the oxidation of unburnt carbon [54]. The mineralogy of the raw biomass ash was determined with X-ray diffraction (XRD) and is given in Figure 1. The ash was found to be rich in polysilicates, namely quartz (SiO_2_), magnesium silicate (MgSiO_3_), wollastonite (CaSiO_3_), and akermanite (Ca_2_Mg(Si_2_O_7_)). A small fraction of calcite (CaCO_3_) was also present in the raw material. Images of the raw biomass ash as received and hand ground, in addition to a scanning electron microscopy (SEM, Hitachi SU3900, Tokyo, Japan) image showing the morphology of the biomass ash particles, are shown in Figure 2A.

### 2.2. Mechanochemical Activations and Accelerated Carbonation

The mechanochemical activation of the biomass ash was performed in the Retsch PM 100 planetary ball mill. A stainless steel jar of 125 mL capacity containing 30 stainless steel balls 10 mm in diameter, with a total ball mass of 120 g, was used for the milling. The mechanochemical activation conditions of the biomass ash were selected based on previous work [54], in which the CO_2_ uptake of the same biomass ash activated under different milling conditions was examined. An optimal CO_2_ uptake after 24 h of carbonation was found when the total ball mass to BBA mass ratio was 5, the water content was 5% of the BBA, and the milling duration was 20 min. The milling speed was set at 500 rpm in all conditions. Thus, these conditions were applied to prepare the mechanochemically activated biomass ash (MBA) used in this study. The quantities of the materials used for one milling are shown in Table 1.

After milling, the mechanochemically activated biomass ash was subjected to accelerated carbonation. First, the milled material was placed in weight trays containing 1.5 g of MBA each so that a thin layer of material was spread on the surface of the trays. The MBA was then carbonated for 24 h in the LABLINE 490-IBRC3 CO_2_ incubator in which the CO_2_ concentration was 20% *v/v* and the pressure was ambient (atmospheric). The relative humidity and the temperature in the incubator were kept at 60 ± 3%, and 22 ± 1 °C, respectively. After 24 h, the carbonated MBA (cMBA) was removed from the CO_2_ incubator and kept in sealed plastic bags at 25 ± 2 °C. The CO_2_ uptake was estimated in the previous work to be 3.9 g of CO_2_ per 100 g of mechanochemically treated biomass ash [54].

The mineralogy of the raw biomass ash (BA), the mechanochemically activated (MBA), and the carbonated mechanochemically activated (cMBA) biomass ash is given in Figure 1, where the XRD spectra of the respective materials are presented. The mechanochemical activation led to a decrease of the intensity of akermanite and the formation of the carbonate phase baylissite (K_2_Mg(CO_3_)_2_·4H_2_O). The formation of new carbonates in the MBA is attributed to the formation of fractured surfaces that occur upon mechanochemical activation and which react with the atmospheric CO_2_ during the milling and the sample-handling process [59]. Previous work also demonstrated the amorphization of calcite and the increase in amorphous silica content in the mechanochemically treated biomass ash [54]. The accelerated carbonation of the mechanochemically activated biomass ash resulted in the increase in calcite content, while at the same time, the peaks of wollastonite, magnesium silicate, and akermanite were decreased. The increased carbonate content in the cMBA is related to the destruction of Si-O-Si bonds of the polysilicates that are present in the ash upon the mechanochemical activation and subsequently to the increase in active oxygen sites that are available to bind more CO_2_ [58]_._

### 2.3. Sample Preparation

Blended cement pastes were prepared using biomass ash as supplementary cementitious material in three forms—as received (BA), mechanochemically activated (MBA), and carbonated mechanochemically activated (cMBA)—to replace 20% and 40% of the cement clinker by mass. Reference samples of plain cement paste (REF) were also prepared as a benchmark, for which Portland cement CEM I 42.5R and distilled water were used for the preparation of all samples, with a constant water to binder (cement + biomass ash) ratio of 0.5. The mix design of the paste types prepared for this study is shown in Table 2. The pastes were cast in 15 mL centrifuge tubes and were kept well sealed until the day of analysis (XRD, FTIR, TGA). Prior to testing, the hydration of the pastes was arrested at 7 and 28 days using the solvent exchange method described by Calabria-Holley et al. [60]. Briefly, paste samples were crushed into pieces of approximately 1–4 mm, which were subsequently immersed in isopropanol for 24 h. Next, the samples were vacuum-dried in a desiccator for another 24 h. Before testing, samples were ground into fine powder with pestle and mortar.

Cement mortar samples were prepared only for the highest substitution (40% replacement) of cement with mechanochemically activated (MBA) and carbonated (cMBA) biomass ash. Standard sand complying with BS EN 196-1 was used, and the mortars were hand-mixed. The mix design followed that of the prepared pastes, including the sand, and is given in Table 3. Six samples per mix design were cast in cylindrical moulds 60 mm in height and 30 mm in diameter. All samples were demoulded 24 h after casting and were subsequently cured in water at 20 ± 1 °C until the day of testing under compression. The cylindrical mortar samples prepared for the examined sample types are shown in Figure 2B. The colour of the biomass-ash-modified mortars appeared darker due to the addition of the dark-coloured biomass ash.

### 2.4. Testing Methods

#### 2.4.1. Hydration Kinetics

The effect of the partial cement replacement with the examined biomass ash types on the hydration kinetics of the cementitious composites was studied with isothermal calorimetry. The control paste was prepared using 20 g of cement and a water-to-cement ratio of 0.5. Pastes with biomass ash (BA, MBA, and cMBA) at 20% and 40% replacement ratio (by mass) using a water-to-binder (cement and biomass ash) ratio of 0.5 were prepared. The tests were carried out with the I-Cal 4000 HPC isothermal conduction calorimeter. Distilled water was added to cement and biomass ash mixes, and the generated pastes were hand-mixed for a maximum of 60 s before being placed in the calorimeter unit for a period of 72 h. The temperature in the unit was set to 20 °C throughout the test.

#### 2.4.2. X-ray Diffraction (XRD)

The powder XRD was conducted with a STOE STADI P (Cu radiation, λ = 1.54 Å) instrument in transmission mode, operated at 40 kV and 40 mA. The diffraction results were recorded from 5° to 75° (2θ), with a step resolution of 0.015° (2θ) per step. A double Mythen detector was used, and each detector covered a range of 19° (2θ) and operated at 31.6 s per degree (2θ).

#### 2.4.3. Attenuated Total Reflectance Fourier Transform Infrared (ATR-FTIR) Spectroscopy

ATP-FTIR was conducted with the Perkin Elmer Frontier instrument using the transmission cell. The transmission spectra were recorded from wavenumber 400 cm^−1^ to 4000 cm^−1^, with 16 repeated scans at the resolution of 1 cm^−1^ for each spectrum acquisition.

#### 2.4.4. Thermogravimetric Analysis (TGA)

The TG analysis was performed with the Netzsch Sta 449F1 Jupiter machine with a heating program ramped from room temperature to 950 °C at a constant heating rate of 10 °C/min under an inert N_2_ gas atmosphere with a gas flow of 60 mL/min.

#### 2.4.5. Compressive Strength

Mortar samples were tested for compressive strength at 7 and 28 days. Instron 50 kN and 100 kN hydraulic frames were used for testing the 7-day- and 28-day-old samples, respectively. A triplet of samples was tested per mix design, and their final strength values were occurred from their average.

## 3. Results and Discussion

### 3.1. Hydration Kinetics

**Figure 3:**
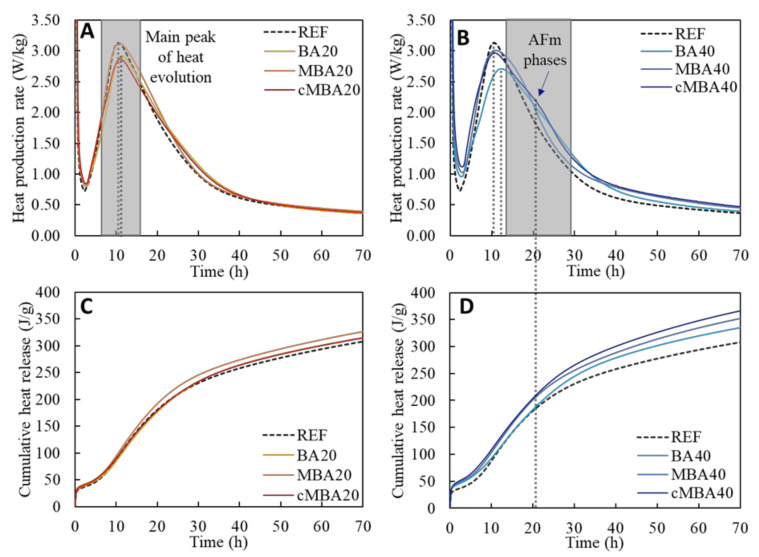
Heat flow rate (**A**,**C**) and accumulated heat release (**B**,**D**) of blended cement pastes prepared with biomass ash (untreated: BA, mechanochemically treated: MBA, and carbonated mechanochemically treated: cMBA) at 20% (**A**,**B**) and 40% (**C**,**D**) cement replacement levels.

The heat production rate and the cumulative heat release during the hydration of the examined samples are presented in Figure 3A,C, respectively, for reference (REF), as are those of blended cement samples with 20% replacement of cement with biomass ash (BA20, MBA20, and cMBA20). In Figure 3B,D, the respective results for the samples with 40% replacement of cement by biomass ash (BA40, MBA40, and cMBA40) are shown along with the REF for comparison.

In all samples, a rapid initial heat release was observed in the first couple of minutes of hydration, immediately after the contact of the dry materials with water. The exothermic reaction taking place at this initial stage is attributed to the dissolution of the unhydrated silicate and aluminate phases (mainly C_3_S and C_3_A) and the formation of ettringite (AFt) that occurs upon the reaction of C_3_A with the sulphate present in the solution [61]. A slowdown of the heat rate was then noticed until approximately the second hour of hydration, during the induction period. This was followed by the acceleration period, at the end of which the main peak of heat evolution is achieved. The hardening of the pastes started during this acceleration period, which is associated with the formation of the main hydration products, i.e., calcium silicate hydrate (C-S-H) and portlandite (CH) [62]. As shown in Figure 3A,B, the main peak of heat evolution occurred after 10.5 h in the reference sample. The replacement of cement with untreated biomass ash by 20% and 40% (BA20 and BA40 samples) resulted in a delay of approximately 2 h in the occurrence of the main hydration peak, which was reached after 11.6 and 12.5 h, respectively. Prolonged induction periods upon the addition of biomass ash are expected according to the literature and are related to the lower cement content and its replacement with a less reactive material [17]. Contrarily, the induction period was not significantly affected in the samples containing the mechanochemically treated and the carbonated mechanochemically treated biomass ash. More specifically, the main peak of hydration in the MBA20 samples occurred after 10.7 h and in the MBA40 after 11.1 h, while the times for the cMBA20 and cMBA40 were 11 and 10.6 h, respectively. The enhanced hydration of cement upon the addition of the mechanochemically activated biomass ash is compared to that of untreated biomass ash. High-impact milling results in materials of smaller particle size, lower crystallinity, and higher surface area, improving their role as fillers in the composite, partly compensating for the dilution effect [63,64]. The effect of carbonation was different in samples of 20% and 40% replacement ratio. It appears to counteract the positive effect of mechanochemical activation in samples with a 20% substitution level, whereas it did not appear to significantly affect the samples with a 40% substitution level.

The hydration peak of all samples was followed by the deceleration period. A discrete shoulder peak centred at 20 h was noticed in the cMBA40 pastes (Figure 3B), whilst it was not obvious in the reference or the other blended pastes. This peak is correlated with the reaction of aluminate phases and the depletion of sulphate to produce ettringite and monosulphate or monocarbonate (AFm) phases [12,65]. Thus, it is suggested that the amplified filler effect of the mechanochemically treated biomass ash promoted the nucleation of aluminate hydrates [12]. The higher heat evolution of the biomass-ash-modified pastes at 40% substitution after the 30th hour of hydration (Figure 3B) could also indicate a higher reactivity of the biomass ash in these samples [62].

The increased cumulative heat release in the samples containing biomass ash at a high substitution level (Figure 3D) is primarily associated with the higher water-to-cement ratio in these samples [61]. Since the heat release of the cMBA40 pastes is further increased after the 20th hour, it can also be correlated with the enhanced aluminate hydration in these samples considering the high enthalpies of the relevant reactions [61].

### 3.2. Phase Assemblages

**Figure 4:**
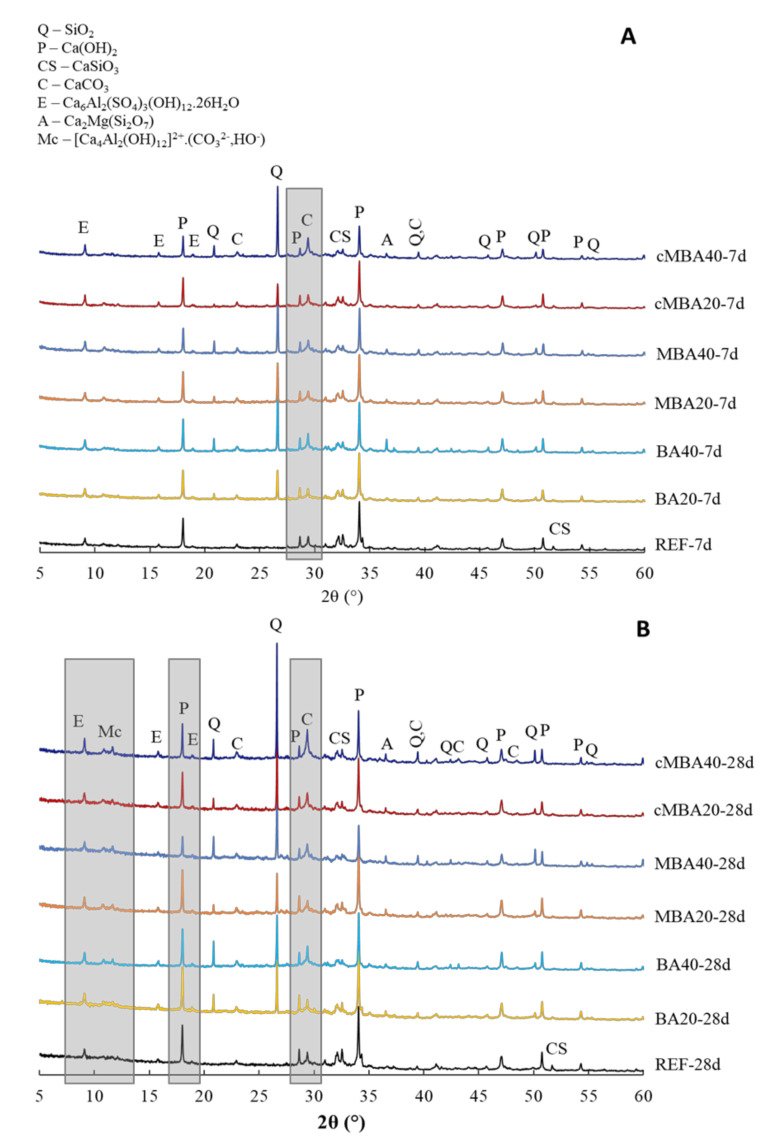
XRD of (**A**) 7-day and (**B**) 28-day blended cement pastes prepared with biomass ash (untreated: BA, mechanochemically treated: MBA, and carbonated mechanochemically treated: cMBA) at 20% and 40% cement replacement levels. Q: quartz, P: portlandite, C: calcite, CS: calcium silicate, C: calcite, E: ettringite, A: akermanite, Mc: monocarbonate and hemicarbonate (AFm phases).

The mineralogy of blended cement pastes with the three types of biomass ash (BA, MBA, and cMBA) substituting 20% and 40% of cement by mass was examined with XRD at 7 (Figure 4A) and 28 days (Figure 4B) of curing. The presence of hydration products, namely portlandite (Ca(OH)_2_, PDF#04-733) and ettringite (Ca_6_Al_2_(SO_4_)_3_(OH)_12_∙26H_2_O, PDF#37-1476), as well as unhydrated calcium silicate (CaSiO_3_, PDF#34-0558) were observed in the 7 days reference and blended pastes. C-S-H (Ca_1.5_SiO_3.5_·xH_2_O, PDF#33-0306) can also possibly be identified. However, because its main peaks at 29.30°, 32.06°, and 50.03° (2θ) overlap with the peaks of the more crystalline calcite and quartz, it was omitted from the identified phases in Figure 4A,B.

Due to the silica-rich biomass ash used in this study, quartz (SiO_2_, PDF# 46-1045) was present in all blended pastes at 7 days, with its peak being more prominent in the samples containing higher amount of biomass ash (BA40, MBA40, cMBA40). Akermanite (Ca_2_Mg(Si_2_O_7_), PDF#77-1149) was observed from the raw biomass ash and was also identified in the blended paste samples. As discussed in Section 2.2, the peak of akermanite decreased upon the mechanochemical treatment of biomass ash, which explains the lower peak of this phase in the MBA and cMBA blended pastes. A low fraction of calcite (CaCO_3_, PDF#05-0586) was detected in the reference as well as in the blended pastes. A small amount of limestone (<5%) is a common addition in Portland cements, explaining the calcite peak of the reference samples. Additionally, the biomass ash used in this study contains a low fraction of calcite, as shown in Section 2.2, thus its presence is expected in the blended pastes as well. The intensity of the calcite peak was higher in the samples containing the carbonated biomass ash at 40% cement replacement, indicating an increased calcite content in the carbonated mechanochemically activated biomass ash used in these samples.

At 28 days, the reference paste presented similar phases to the sample at 7 days, showing only a slight increase in the intensity of the hydration products (portlandite, ettringite). On the other hand, in all blended pastes of this age, AFm phases (hemi-carbonate at 10.8° and mono-carbonate at 11.7°) were detected. These were more prominent in the cMBA40 paste, in which the ettringite peak also appeared to be more pronounced. This is in line with the calorimetry results, which showed increased hydration of the aluminate phases in this sample. Hemicarbonate and monocarbonate are commonly formed in blended cements in the presence of limestone and are often accompanied by ettringite stabilisation [66]. The particularly low portlandite peaks of the MBA40 paste at 28 days, which was not noticed at 7 days old pastes, could suggest that the formation of additional aluminate hydrates in these samples was the result of the slow pozzolanic reactivity of the mechanochemically activated biomass ash in addition to its filler effect. Similar observations of increased AFm phases were made by Skocek et al. [67], in whose study the pozzolanic reaction of carbonated cement fines was studied. Nonetheless, the qualitative analysis of the XRD results does not allow definite conclusions on this. Finally, the decrease in the unhydrated calcium silicate peak at 52° (2θ) noticed in all blended pastes as compared to the reference is related to the higher effective water-to-cement ratio in these pastes and hence the complete hydration of the available cement in these samples [65].

**Figure 5:**
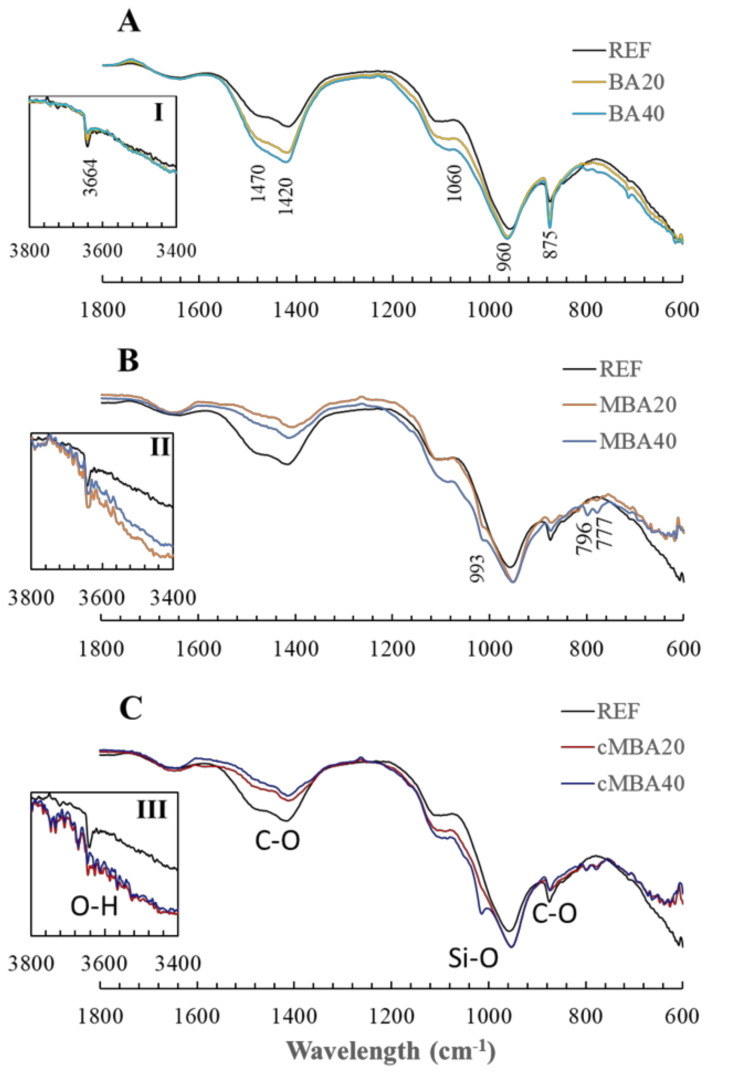
FTIR of 28−day blended cement pastes prepared with (**A**): untreated biomass ash, (**B**): mechanochemically activated biomass ash, and (**C**): carbonated mechanochemically treated biomass ash at 20% and 40% cement replacement levels.

The chemical bonds formed in the reference and the biomass ash blended pastes were studied with FTIR, and the respective spectra are presented in Figure 5. The wavelength at which the peaks occurred are given in Figure 5A, whereas the additional peaks noticed only in the samples containing the mechanochemically activated biomass ash are noted in Figure 5B. The assignment of major vibration bonds identified in these samples is shown in Figure 5C. The bands at 1420 and 1470 cm^−1^ correspond to the asymmetrical stretch of C-O bonds [68], indicating the presence of crystalline calcite [68] and other CaCO_3_ polymorphs [68] or poorly crystallised carbonates [54], respectively, in all examined pastes. The peak at 875 cm^−1^ is also characteristic of calcite, resulting from the asymmetrical bending of C-O bonds [68,69].

The peaks that appear between 700 and 1200 cm^−1^ are attributed to the vibration of silica bonds (Si-O) [68]. XRD analysis revealed the presence of quartz as well as silicates such as akermanite in the reference and in the blended cement pastes. Thus, it is expected that Si-O vibrations of these phases will occur in this wavelength range. More specifically, the peak that occurred at 960 cm^−1^ is related to the stretch of Si-O bonds in Q^2^ silica, while the peak at 1060 cm^−1^ is also associated with bond vibrations in Q^4^ amorphous silica [70]. These peaks were present in all samples. In the mechanochemically treated samples specifically, both carbonated and noncarbonated, the occurrence of the double band at 796 and 777 cm^−1^ indicates the presence of quartz [68]. An additional peak at 993 cm^−1^ related to the stretching vibration of Si-O bonds [67,69] appeared only in the samples containing the mechanochemically treated biomass ash (shown in Figure 5B,C), suggesting that additional silicate hydrates were present in these samples [71]. FTIR, contrarily to XRD, allows the detection of amorphous phases [68]. Thus, it possible that C-S-H phases were formed in the mechanochemically activated as a result of the filler effect and pozzolanic reactivity, which were enhanced in these samples.

Figure 5(I–III) provide a focused view of the band occurring at 3664 cm^−1^, which is caused by the stretching of O-H bonds and is characteristic of portlandite [68]. The presence of portlandite is thus evident in the reference and the BA20 and BA40 samples. The intensity of this peak is lower in the BA20 and BA40 samples, which could be related to the dilution effect in these samples and hence the formation of fewer hydration products, including portlandite, corresponding to the hydration kinetics analysis (Section 3.1). A smaller peak of portlandite is also noticed in the pastes containing the mechanochemically activated biomass ash (Figure 5(II,III)), which did not result in higher carbonate content in the samples. It is possible that part of the portlandite was consumed in the pozzolanic reaction, reacting with the active silicates that were induced in the biomass ash upon the mechanochemical treatment [63] and forming the additional silicate hydrates noticed in these samples.

**Figure 6:**
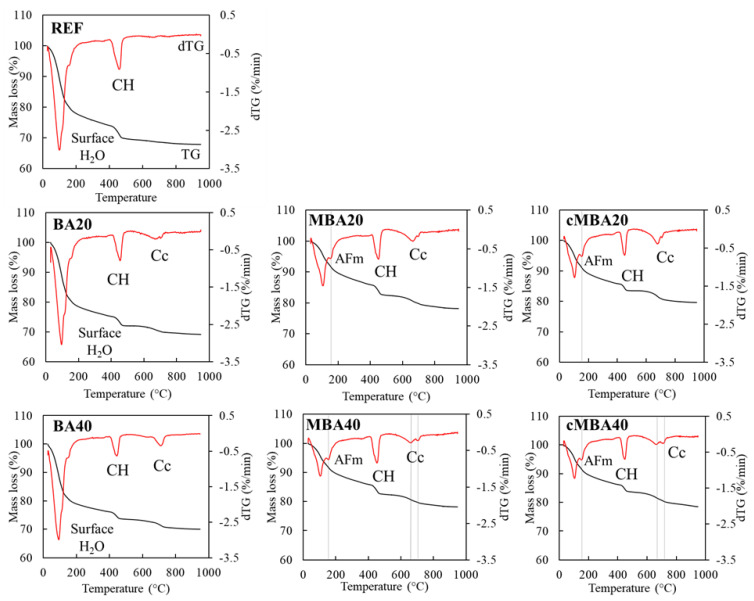
Thermalgravimetric analysis of 28-day samples blended cement pastes prepared with biomass ash (untreated: BA, mechanochemically treated: MBA and carbonated mechanochemically treated: cMBA) at 20% and 40% cement replacement levels. CH: portlandite, Cc: calcium carbonate, AFm: AFm phases (mono- and hemicarbonate).

The thermogravimetric analysis of the 28-day-old examined samples is shown in Figure 6, in which the mass loss over temperature (TG) and their derivative curves (dTG) are presented. A high loss of surface water is observed in the REF, BA20, and BA40 samples shown, with a pronounced peak at around 100 °C. Since the XRD and FTIR analyses did not reveal the presence of additional hydration products in these samples, it is considered that this extensive mass loss of water is associated with the exposure of these samples to moisture prior to testing. The second peak that occurs between 400–500 °C in all samples corresponds to the dehydroxylation of portlandite. In the temperature region of 600–800 °C, mass loss due to decarbonisation of carbonates is noticed in all blended pastes but not in the reference.

The dTG peak of decarbonisation appeared to be broad, extending from approximately 550 °C to 720 °C in all samples, except for BA40, for which a more well-defined peak was noticed. In MBA40 and cMBA40, a double decarbonation peak could be detected centred at around 660 °C and 700 °C. The two-step decomposition of carbonates suggests the presence of different calcium carbonate polymorphs in these materials [72], while the occurrence of decarbonation at temperature lower than 700 °C could be associated with the formation of less crystalline calcium carbonate [73]. A mass loss peak at around 180 °C was detected only in the samples for which mechanochemically activated biomass ash was used (both uncarbonated and carbonated), confirming the presence of AFm phases in these samples [67], as reported earlier from the hydration kinetics and the XRD analysis.

**Figure 7:**
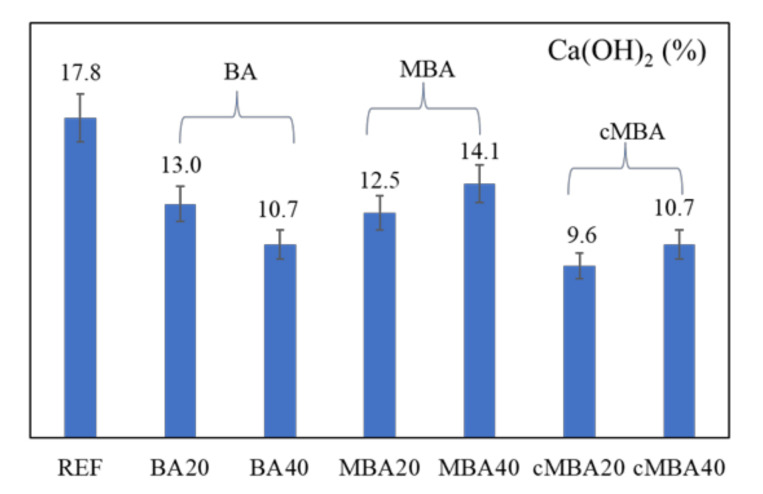
Portlandite content of 28-day blended cement pastes prepared with biomass ash (untreated: BA, mechanochemically treated: MBA, and carbonated mechanochemically treated: cMBA) at 20% and 40% cement replacement levels. Error bars present a relative error of 7.5%.

Portlandite content in the examined samples was quantified based on the TGA curves using Equation (1) [74], and the results are presented in Figure 7. A relative error of ±7.5% due to the heterogeneity of the paste and the small amount of the samples used for the analysis (0.05 g) was considered [74].

(1) $$\mathrm{Ca}{\left(\mathrm{OH}\right)}_{2}={\mathrm{WL}}_{\mathrm{Ca}{\left(\mathrm{OH}\right)}_{2}}\times \frac{m_{\mathrm{Ca}{\left(\mathrm{OH}\right)}_{2}}}{m_{H_{2}O}}$$

where ${\mathrm{WL}}_{\mathrm{Ca}{\left(\mathrm{OH}\right)}_{2}}$: mass loss due to dehydroxylation of Ca(OH)_2_, and $m_{\mathrm{Ca}{\left(\mathrm{OH}\right)}_{2}}$, $m_{H_{2}O}$: molecular masses of the noted compounds, equal to 74 and 18 g/mol, respectively.

Figure 7 shows that all 28-day blended cement pastes had lower portlandite content than the reference (17.8%). The lower portlandite content in the pastes containing the untreated biomass ash (BA) could be related to the formation of fewer hydration products in these samples as a result of isothermal calorimetry due to the dilution effect. In addition, FTIR and TG analysis demonstrated a higher calcium carbonate content in these samples, which also explains the decrease of portlandite. It is likely that the replacement of cement with the untreated biomass, a material coarser than cement, led to an increased porosity in these samples, which could facilitate their carbonation at 28 days of age compared to the reference samples. On the other hand, the quantity of portlandite was slightly higher in the samples in which cement was replaced with the mechanochemically treated biomass ash (MBA) due to the increased filler effect of the treated biomass ash, as demonstrated from the hydration kinetics analysis. It is also possible that some portlandite was consumed in the pozzolanic reaction for the formation of more silicate hydrates in these samples, as noticed in the FTIR analysis. Finally, the lowest portlandite content noted in the carbonated mechanochemically activated biomass ash (cMBA) can be related both to the dilution effect and the increased pozzolanic activity of the ash, consistent with the result of the FTIR.

### 3.3. Strength Performance

**Figure 8:**
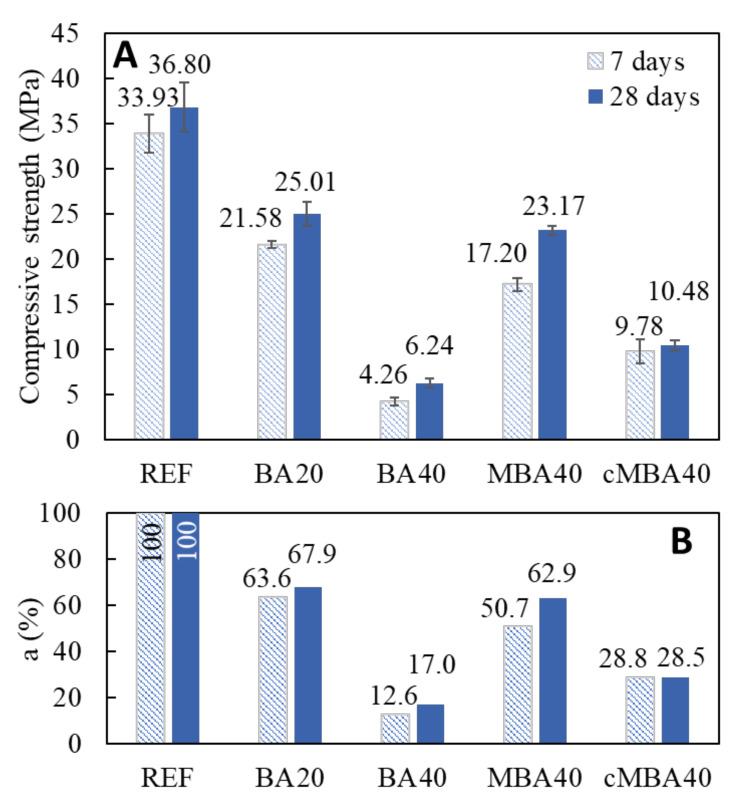
(**A**) Compressive strength at 7 days and 28 days of the reference and the blended cement mortars prepared with biomass ash at 20% and 40% cement replacement levels and (**B**) their relative percentages (%) in comparison to the average strength performance of REF sample at 7 days and 28 days, respectively. BA: untreated; MBA: mechanochemically treated; cMBA: mineral carbonated after mechanochemical treatment. Error bars present standard deviation.

Figure 8 shows the 7- and 28-day compressive strength results of the examined blended cement mortars as well as the respective percentages (a%) of the reference strength developed in the blended mortars. The results showed a decline in strength for all blended cement samples caused by the substitution of Portland cement with the various types of biomass ash. The substitution of cement with untreated biomass ash by 20% led to the development 63.6% and 67.9% of the reference mortars’ compressive strength at 7 and 28 days, respectively. Doubling the substitution level further impaired the strength, resulting in the development of only 12.6% and 17% of the references’ strength in the 7- and 28-day-old BA40 samples, respectively. Significant compressive strength decline at such high substitution levels of cement with BBA has been previously reported by Rosales et al., who found that only 36% of the strength developed in mortars with 38.5% cement replacement [29]. The higher strength loss noticed in this study can be related to the specific BBA used and the combustion conditions from which it derived. As noticed by Ottosen et al. [27] biomass ash deriving from small burning facilities was reported to lead to higher strength losses in blended cement composites.

The addition of mechanochemically treated biomass ash, however, even at the high level of 40% cement replacement, resulted in a less pronounced strength impairment. The strength reached 50.7% and 62.9% of the reference’s strength in the 7- and 28-day-old MBA40 samples, which signifies an increase of 270% compared to the strength of the BA40 samples. As a result, the high replacement ratio in the MBA40 samples led to similar strength performances of the BA20 samples containing half the amount biomass ash. The positive effect of the mechanochemical treatment on the reactivity of the biomass ash is therefore reflected in the strength results. An enhanced filler effect of the mechanochemically activated biomass could have contributed to the strength enhancement of the MBA40 samples as compared to the BA40 by providing more surfaces to enable the precipitation of reaction products and promote the formation of denser microstructures. Thus, the formation of hydration products in these samples could have partly compensated for the dilution effect due to the significant reduction of the cement clinker. At the same time, the destructed Si-O-Si bonds on the surface of silicate minerals due to the high impact milling and the subsequent increase of the surface free energy could have enhanced the pozzolanic reactivity of the biomass ash [63,64]. Similar results have been reported by [23], who found that mechanochemical activation improved the reactivity of the biomass fly ash, resulting in strength comparable to that of the reference for 5–15% cement replacement. The combination of mechanochemical activation with other treatments that have been reported to enhance the performance of biomass ash in cement-based materials, such as the removal of lightweight particles by floatation [29] and the addition of silica fume during the milling process [38], is worthy of further investigation for obtaining higher strength composites.

The replacement of 40% of the cement with the carbonated mechanochemically activated biomass (cMBA40) resulted in the development of 28.8% and 28.5% of the reference’s compressive strengths at 7 and 28 days, respectively, which were much lower values than those of the MBA mortars but still almost double those of BA mortars. The carbonation process led to the formation of stable carbonate phases in the mechanochemically activated biomass ash. Thus, the active Si-O bonds on the surface of the mechanochemically treated biomass ash were possibly bound with CO_2_ after the carbonation treatment, leaving fewer active sites in the cMBA compared to the MBA. The formation of carbonates could have also resulted in higher particle sizes of the cMBA, which would again reduce their filler effect compared to the MBA.

## 4. Conclusions

This study examined the hydration kinetics, phase assemblages, and mechanical properties of blended cement composites prepared by partial substitution of cement clinkers with biomass ash treated with mechanochemical activation and accelerated carbonation. The results showed that:

1. The mechanochemical treatment had a positive effect on the hydration of biomass ash-cement composites, resulting in shortened induction periods compared to the untreated biomass ash.
2. The 28-day strength performance of mortars containing the mechanochemically activated biomass ash at high substitution level (40%) was improved by approximately 270% compared to that of mortars containing the same amount of untreated biomass ash. The 40% substitution of cement with mechanochemically activated biomass ash led to similar strength performance of 20% substitution with untreated biomass ash.
3. On the other hand, the carbonation of the mechanochemically treated biomass ash resulted in lower strength of the respective mortar samples, which, nevertheless, was still higher by 67% than that of samples prepared with the untreated biomass ash.

The results suggest that mechanochemical activation can be used to improve the reactivity and the filler effect of non-reactive alkaline solid wastes, such as the biomass bottom ash used in this study. The use of activated BBA at the high replacement ratio of 40% used in this study can only be realised in non-structural low-strength concrete applications, such as backfilling and road bedding. At lower substitution levels, close to 15–20%, the mechanochemically activated biomass ash is expected to have little to no adverse effect on the strength performance of mortars. It is, therefore, a method that could broaden the range of the locally available industrial byproducts that can be utilised in cement-based materials. In addition, it could further enhance the reactivity of alkaline solid wastes that are already used as SCMs and hence increase their substitution levels in cementitious composites without compromising the strength performance. Although the carbonation of biomass ash prior to its incorporation in cementitious composites can reduce the carbon footprint of the composite to net zero, the substitution levels should be kept relatively low, close to 5–10%, to avoid significant strength reductions. Further investigation on the durability properties of these low-clinker composites is necessary to draw conclusions on the suitability of their use. Finally, mechanochemical activation and carbonation conditions can be further improved to achieve an optimum balance of improved CO_2_ uptake and enhanced reactivity in biomass ash and other alkaline solid wastes.

## Figures and Tables

**Table 1:** The mass quantities of the materials used per milling.

Sample ID	Total Mass of 30 Stainless Steel Balls (g)	BA (g)	Distilled Water (g)
MBA	120	24	1.2

**Table 2:** Mix design of all paste samples per 100 g of cement. BA: untreated biomass ash; MBA: mechanochemically treated biomass ash; cMBA: carbonated mechanochemically treated biomass ash.

Sample ID	CEM I (g)	BA (g)	MBA (g)	cMBA (g)	Sand (g)	Water (g)
REF	100	0			300	50
BA-20	80	20			300	50
BA-40	60	40			300	50
MBA-20	80		20		300	50
MBA-40	60		40		300	50
cMBA-20	80			20	300	50
cMBA-40	60			40	300	50

**Table 3:** Mix design of all mortar samples per 100 g of cement. BA: untreated biomass ash; MBA: mechanochemically treated biomass ash; cMBA: carbonated mechanochemically treated biomass ash.

Sample ID	CEM I (g)	BA (g)	MBA (g)	cMBA (g)	Sand (g)	Water (g)
REF	100	0			300	50
BA-20	80	20			300	50
BA-40	60	40			300	50
MBA-40	60		40		300	50
cMBA-40	60			40	300	50

## Data Availability

Data will be made available on request and are also accessible through the University of Bath’s research portal.
